# The miniature genome of a carnivorous plant *Genlisea aurea* contains a low number of genes and short non-coding sequences

**DOI:** 10.1186/1471-2164-14-476

**Published:** 2013-07-15

**Authors:** Evgeny V Leushkin, Roman A Sutormin, Elena R Nabieva, Aleksey A Penin, Alexey S Kondrashov, Maria D Logacheva

**Affiliations:** 1 Department of Bioengineering and Bioinformatics, Lomonosov Moscow State University, Leninskye Gory 1-73, Moscow 119992, Russia; 2 Institute for Information Transmission Problems of the Russian Academy of Sciences, Moscow 127994, Russia; 3 Department of Genetics, Lomonosov Moscow State University, Moscow 119992, Russia; 4 Department of Ecology and Evolutionary Biology and Life Sciences Institute, University of Michigan, Ann Arbor, MI 48109, USA; 5 A.N. Belozersky Institute of Physico-Chemical Biology, Lomonosov Moscow State University, Moscow, Russia

**Keywords:** Genome reduction, Carnivorous plant, Intron, Intergenic region

## Abstract

**Background:**

*Genlisea aurea* (Lentibulariaceae) is a carnivorous plant with unusually small genome size - 63.6 Mb – one of the smallest known among higher plants. Data on the genome sizes and the phylogeny of *Genlisea* suggest that this is a derived state within the genus. Thus, *G. aurea* is an excellent model organism for studying evolutionary mechanisms of genome contraction.

**Results:**

Here we report sequencing and *de novo* draft assembly of *G. aurea* genome. The assembly consists of 10,687 contigs of the total length of 43.4 Mb and includes 17,755 complete and partial protein-coding genes. Its comparison with the genome of *Mimulus guttatus*, another representative of higher core Lamiales clade, reveals striking differences in gene content and length of non-coding regions.

**Conclusions:**

Genome contraction was a complex process, which involved gene loss and reduction of lengths of introns and intergenic regions, but not intron loss. The gene loss is more frequent for the genes that belong to multigenic families indicating that genetic redundancy is an important prerequisite for genome size reduction.

## Background

In spite of the similarity of basic cellular processes in eukaryotes, their genome sizes are extraordinarily variable. The question “Why are some genomes really big and others quite compact?” was listed by Science as one of 125 big questions that face scientific inquiry over the next quarter-century. Flowering plants provide an excellent opportunity to address this question [1]. A monocot *Paris japonica* possesses a 150,000 Mb genome, the largest genome known [2]. By contrast, two carnivorous plants from the family Lentibulariaceae, *Genlisea margaretae* and *G. aurea* have genomes of only 63.4 Mb and 63.6 Mb, respectively, although genomes of some other species of these genus exceed 1,000 Mb [3]. Such flexibility of the genome size is of interest from both the evolutionary and functional points of view. In a model plant species, *Arabidopsis thaliana*, number of protein-coding genes is 27416 (TAIR 10) and average gene length is about 2,300 bp [4,5]. This gives an estimate of genic (coding + introns + untranslated regions) fraction length close to 60 Mb. If Arabidopsis-based estimates were applicable to small-genome *Genlisea* species, this would imply that they either lost a large proportion of their genes or possess only very short intergenic regions.

Recent advances of sequencing technologies made it possible to characterize genomes of a number of angiosperm species. Most of them are from economically important species (such as rice, potato, soybean and apple). Also, the genome projects for plants of outstanding evolutionary significance such as basal angiosperm *Amborella* and basal eudicot *Aquilegia* are in progress [6-8]. Together with availability of efficient tools and databases for plant genome annotation [9-11], this enables studies of the genome size evolution in angiosperms. Phylogeny of genus *Genlisea*[12] implies that the small genomes of closely related *G. margaretae* and *G. aurea* is a derived condition, because the genomes of both their sister species *G. hispidula* (1,510 Mb), and outgroup species *G. violacea* (1,005 Mb), *G. lobata* (1,227 Mb), and *G. uncinata* (995 Mb) are much larger [3]. This indicates that after its divergence from the *G. hispidula,* genomes in small-genome *Genlisea* lineage underwent contraction by the factor of more than 10.

A variety of mechanisms can be responsible for genome contraction in the evolutionary history of *Genlisea*. First, large genome segments, perhaps even full chromosomes, could be lost. However, this is unlikely to be the case: genomes of *G. margaretae* and *G. aurea* consist of more chromosomes than *Genlisea* species with bigger genome size (40–52 vs 22–32) [3]. Second, loss of genes could be involved. Whole-genome duplications (WGD) occurred several times during the diversification of angiosperms, leading to massive emergence of paralogous genes. Though functional divergence of duplicated genes is an important mechanism in plant evolution, in some cases the paralogs are completely or partially redundant, thus being plausible candidates for gene loss [5]. Third, a significant part of plant genome is represented by repetitive DNA (up to 80% in grasses and conifers [13,14]) and apparently could be reduced. Fourth, shrinkage of non-coding DNA is also possible: though parts of intergenic regions or introns are functionally important and have to be retained in evolution [15-17], a large proportion of non-coding sequences appear to be dispensable.

Recently, the genome of *Arabidopsis lyrata*, a close relative of model plant species *A. thaliana*, has been sequenced [18]. *A. lyrata* genome is about 1.7 times larger than *A. thaliana* genome. Comparison of these genomes revealed that the difference in their sizes was mostly due to small deletions in non-coding DNA. The gene number in *A. lyrata* is also a little higher than in *A. thaliana*. This suggests that gene losses occurred during the recent evolution of *A. thaliana*, assuming that its small genome is a derived state.

Besides minimization, the genomes of *Genlisea* were reported to have another peculiar feature, an increased rate of nucleotide substitution [19,20]. However, this report is based mostly on the investigation of plastid DNA sequences and only one nuclear gene – that of 5.8S rRNA – was sampled. Recently the increased nucleotide substitution rate was reported for a related species from Lentibulariaceae, *Utricularia gibba*, which also has a small genome [21]. A hypothesis based on the mutagenic action of reactive oxygen species was proposed to explain both high substitution rate and changes in the genome size [22]. Characterization of *G. aurea* genome makes it possible to reassess this hypothesis.

## Results

### *G. aurea* genome: sequencing, assembly and validation

**Figure 1:**
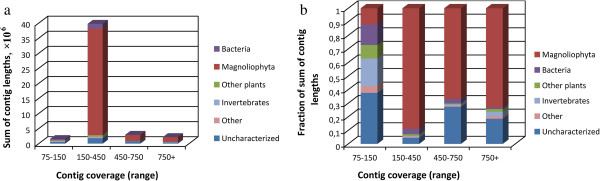
**Total length of *Genlisea aurea *contigs, split into four bins according to the coverage. **For each bin, the total lengths of contigs with the best BLAST hits in different groups of species are shown by color **(A)**. Relative contributions of contigs with the best BLAST hits in different groups of species to the total lengths of contigs with different coverages **(B)**.

Sequencing of the *G. aurea* genome was performed on the Illumina HiSeq2000 machine, using two paired-end libraries with average insert sizes 213 and 423. About 347 millions of paired quality-filtered reads were obtained. The reads were assembled using the CLC Genomics Workbench 5.0.1. The distribution of coverage of the assembly has two modes (Additional file 1). The lower mode is primarily due to contaminating DNA. *Genlisea,* like members of its sister genus *Utricularia*, live in close association with microbial community [22], and, because whole plants were used for DNA extraction, a small amount of DNA from periphyton was also present. To exclude the contigs derived from contaminating organisms we performed a two-step selection based on a read depth and similarity to known flowering-plant sequences. First, following an approach developed in [23] for nonaxenic cultures and the observation that contamination was an important issue mostly for the lower-coverage contigs (Figure 1), we removed contigs which have very low coverage (<75). This resulted in a set of 11,261 contigs covering 46 Mb. Thus, we further filtered this set according to either similarity to the known DNA sequences from *Magnoliophyta* or to the absence of similarity to any known sequences (see Methods). As a result, we obtained the final high-quality set of 10,687 contigs (lengths above 1000 nt) covering 43.4 Mb or 68% of the genome (N50 = 5,786). This proportion is similar to that reported for other plant genome sequencing projects where only (or predominantly) Illumina technology was used [24,25]. Application of the CEGMA pipeline for assessing the resulting gene space [26] showed that 187 or 75.4% of the 248 low-copy Core Eukaryotic Genes are fully present in the assembly, and 230 or 92.7% are present fully or in part. Thus, apparently our assembly covers most of the coding region of *G. aurea* genome. To assess the effects of contamination on the assembly and on our estimates of gene number we performed the test using *Arabidopsis thaliana* sequence data. We assembled *de novo* two sets of reads – “clean”, that contained only *Arabidopsis* reads and “contaminated” that contained also reads from other organisms and then mapped them on reference genome. Among 120 Mbp of all reference chromosomes 22 Mbp (18%) were uncovered in clean dataset and 30 Mbp (25%) were uncovered in contaminated dataset. Among 28,775 genes of reference annotation 3,850 genes (13%) were classified as uncovered in clean dataset and 4,898 genes (17%) were classified as uncovered in contaminated dataset. This shows that, first, contamination has unfavorable effect on assembly, but this effect is not dramatic, second, that non-coding regions are more likely not to be represented in the assembly.

### Transcriptomes of *Utricularia intermedia* and *Pinguicula vulgaris*

In order to gain insight into evolution of the *G. aurea* genome and to improve its annotation with transcriptomic data, we performed cDNA sequencing for two related species, *U. intermedia* and *P. vulgaris*, representing two other genera of the family Lentibulariaceae. About 30 millions of reads of 109 nt in length were obtained for each species (23 and 24 millions after trimming). *De novo* assembly resulted in 40,041 and 42,824 contigs with N50 = 853 and N50 = 1,043 for *P. vulgaris* and *U. intermedia*, respectively. Out of them, 32,096 and 35,752 had significant BLAST hits; taxonomic distribution of best hits is similar to that of *G. aurea* (Additional file 2).

### Characteristics of *G. aurea* genome

The average GC-content of the *G. aurea* genome is 40%. Standard deviation of the GC-content of a contig, 0.064, is much higher than expected under uniform distribution of nucleotides, 0.01 (Additional file 3). There is a negative correlation of intron length with GC-content (ρ = -0.29, Spearman’s test, p < 0.01).

**Figure 2:**
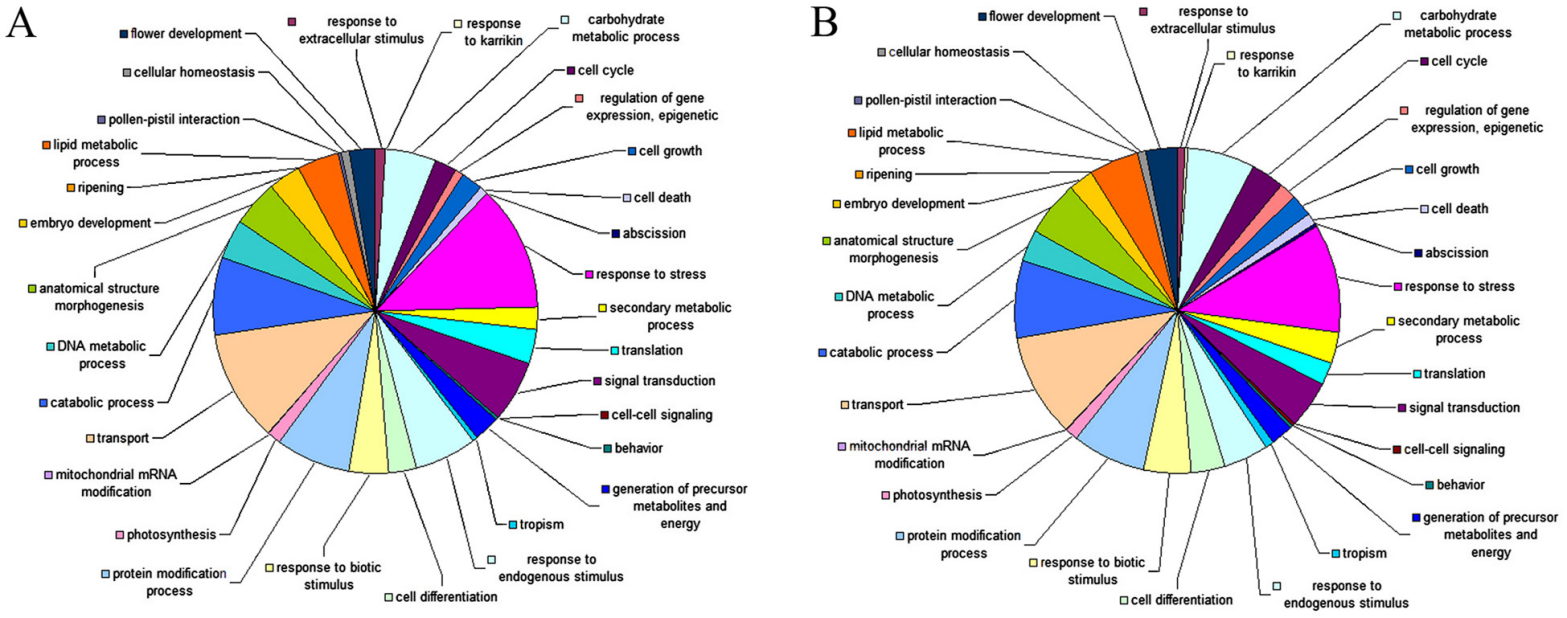
**Distribution of GO-categories for biological process in *Arabidopsis thaliana *(left) and *Genlisea aurea *(right).**

*De novo* gene prediction for the nuclear genome assembly was performed using four different methods (see Methods) and resulted in17,755 gene models (Table 1). Average number of exons per gene is 4.5, average length of gene including introns is 1,433 nt, average transcript length is 965 nt. Out of 17,755 gene models, 15,361 have significant BLAST hits to UniProt with similarity >30%. The species that provided most top-hits is *Vitis vinifera*, followed by *Populus trichocarpa* and *Ricinus communis*. Such taxonomic distribution of top-hit species is similar to that in many other plant transcriptomes. Pfam-domains were found in 13219 proteins. Mean intron length is 134 nt, and median intron length is 89 nt (Additional file 4). Out of 17,755 predicted transcripts, 13,279 were GO-annotated. The distribution of GO-categories in *Genlisea* is similar to *Arabidopsis* (Figure 2). *G. aurea* genome is characterized by a strong codon bias, with the effective number of codons used being equal to 57. GC-content is 55% in third codon position compared to only 34% in intergenic regions.

**Table 1:** **Number of genes predicted in each annotation (aug – AUGUSTUS, gm – GeneMark-ES, gs1 – GENESEQER with *Utricularia intermedia *gs2 - GENESEQER with *Pinguicula vulgaris*, gw1 – GENEWISE with *Mimulus guttatus, *gw2 – GENEWISE with *Arabidopsis thaliana*, gw3 - GENEWISE with *Solanum lycopersicum, *gw4 – GENEWISE with all Uniprot proteins) and number of genes in final dataset**

	**Genes predicted with each method**	**With length more than 50 a.a. and without frameshifts**	**Among all with links to proteomes**	**Genes selected in final dataset**	**With links to uniprot**
aug	11991	11907	9766	1247	848
gm	17245	15590	12625	3675	2180
gs1	11904	11904	11121	1305	1218
gs2	11928	11928	11023	1348	1270
gw1	15122	14915	13761	3260	3129
gw2	14717	14465	13636	2108	2028
gw3	15098	14840	13860	2411	2303
gw4	13689	13324	13277	2401	2385
all				17755	15361

### Comparative genome analysis

**Figure 3:**
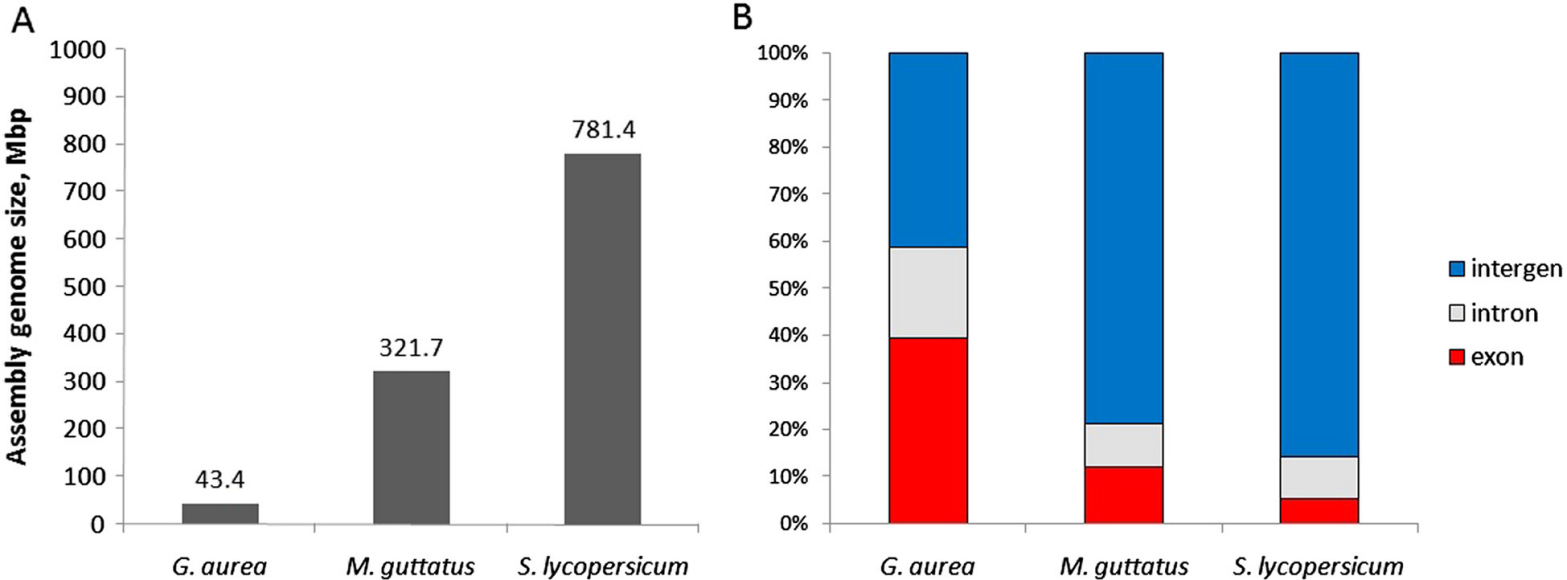
**Genome assembly size (A) and distribution of exon, intron and intergenic sequence lengths for *Genlisea aurea*, *Mimulus guttatus *and *Solanum lycopersicum *(B).**

The closest to *G. aurea* species with known genome is *Mimulus guttatus* (http://www.phytozome.org/mimulus.php). *Mimulus* belongs to the family Scrophulariaceae, which is, together with Lentibulariaceae, a representative of Higher Core Lamiales clade [27]. Thus, in comparative analyses, we used *M. guttatus* genome as a sister to *G. aurea*, and the next closest genome of *Solanum lycopersicum* as an outgroup for this sister pair. Genome assembly sizes and proportions of exonic, intronic and intergenic sequences for these three species are shown in Figure 3. *G. aurea* genome has a 2.4 times smaller total exonic sequence length, 4.0 times smaller total intronic sequence length, and 13.0 times smaller total intergenic sequence length, as compared to *M. guttatus*. As for the number of protein-coding genes, 17,755 were found in our assembly. Even taking into account that 10-20% of genes could have been missed due to incomplete assembly, an estimated total number of genes is much lower in *Genlisea* than in other known angiosperm genomes. *M. guttatus* and *S. lycopersicum* genomes contain 28,282 and 34,257 annotated genes, respectively. Therefore, a smaller number of genes in small genome *Genlisea* lineage is likely due to gene loss, which occurred since its divergence from the *M. guttatus* lineage. To test our hypothesis of dramatic gene loss we performed analysis of the genome regions which are orthologous between *Genlisea* and *Mimulus*. We considered an exon in the *G. aurea* genome to be orthologous to an exon in *M. guttatus* if they satisfy the best bidirectional (TBLASTN-BLASTX) hit criterion. A genome region between two pairs of orthologous exons was also considered as orthologous. We looked for *G. aurea* contigs such that the first and the last gene within them had orthologs in the same *M. guttatus* contig. Then, the gene content of sequence segments between these two pair of orthologs was compared for *G. aurea* and *M. guttatus*. Such segments in *M. guttatus* totally contained 2,801 genes, but for *G. aurea* the corresponding number is only 393. Out of 2,408 *M. guttatus* genes with no collinear ortholog in *G. aurea*, 961 were found to be transposed in *G. aurea* to the other genome regions, whereas 1,477 were apparently lost because either no significant BLAST hit was found or the targeted locus had better similarity to another protein-coding gene in *M. guttatus*. This analysis implies that the observed contraction of the overall length of coding sequences was due to complete loss of a fraction of genes rather than to the shortening of exons. Indeed, there was only a minimal shrinkage of individual genes, as the overall lengths of orthologous exons are very close in the two species: 11.1 Mb for *G. aurea* and 11.3 Mb for *M. guttatus*. Comparison of orthologous intergenic regions reveals shortening similar to contraction observed at the whole-genome level: 673 kb in *G. aurea vs.* 2,744 kb in *M. guttatus* (4.1 times contraction). If we consider only orthologous introns, defined as introns flanked by orthologous exons, their overall lengths are 2.7 Mb in *G. aurea vs.* 6.3 Mb in *M. guttatus*, a 2.4-fold reduction.

**Figure 4:**
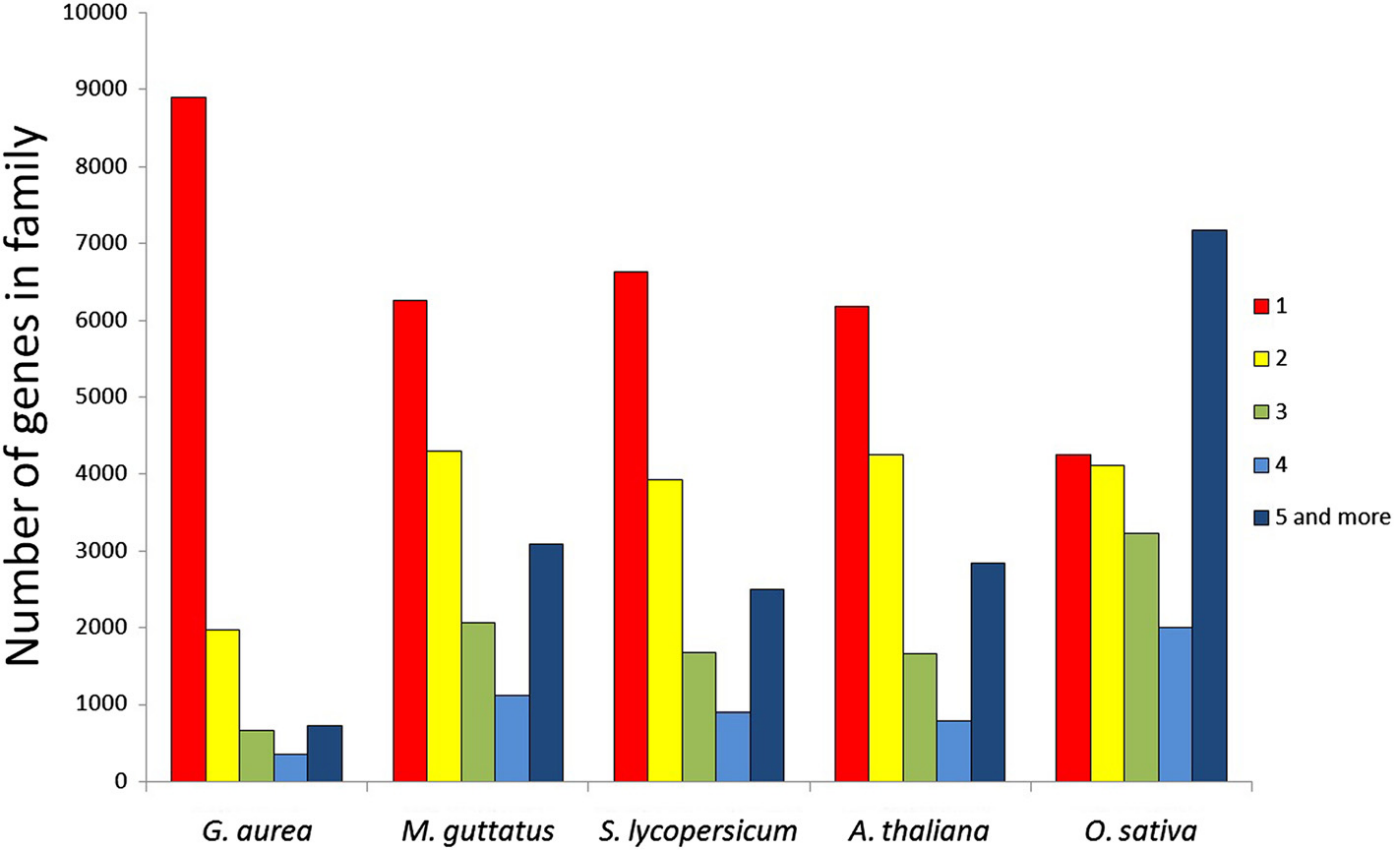
**Number of genes per gene family in *Genlisea aurea *and other plant species as assessed by OrthoMCL.**

Analysis of gene families using OrthoMCL indicates that a gene in *M. guttatus* is more likely to be absent in *G. aurea* if it has paralogs (Additional file 5). Overall, the *G. aurea* genome is biased to genes with lesser number of copies compared e.g. to genomes of *M. guttatus*, *A. thaliana*, *Solanum lycopersicum*, *Oryza sativa* (Figure 4). The analysis of enrichment by specific GO categories revealed that protein kinases which are a large multigenic superfamily - are preferentially lost in the *G. aurea* genome (Additional file 6).

Because the reduction of the genome size in *G. aurea* lineage occurred rapidly, it is natural to assume that fixations of long deletions played a role in this process. Unfortunately, due to large evolutionary distance of *G*. *aurea* and *M. guttatus* their orthologous intergenic regions are hard to align. We were able to do it only using an interactive software tool OWEN [28]. In twenty pairs of randomly chosen intergenic regions, we detected 31 localized length differences longer than 500 nucleotides between the two genomes. These differences are likely due to deletions in the *G. aurea* genome, although insertions in the *M. guttatus* genome also cannot be ruled out, because intergenic regions of the outgroup genome of *S. lycopersicum* are mostly unalignable with both sister genomes and thus do not allow polarization of this character. Six out of these putative deletions were likely associated with direct, low-complexity repeats which could mediate their origin in the ancestral genome.

**Figure 5:**
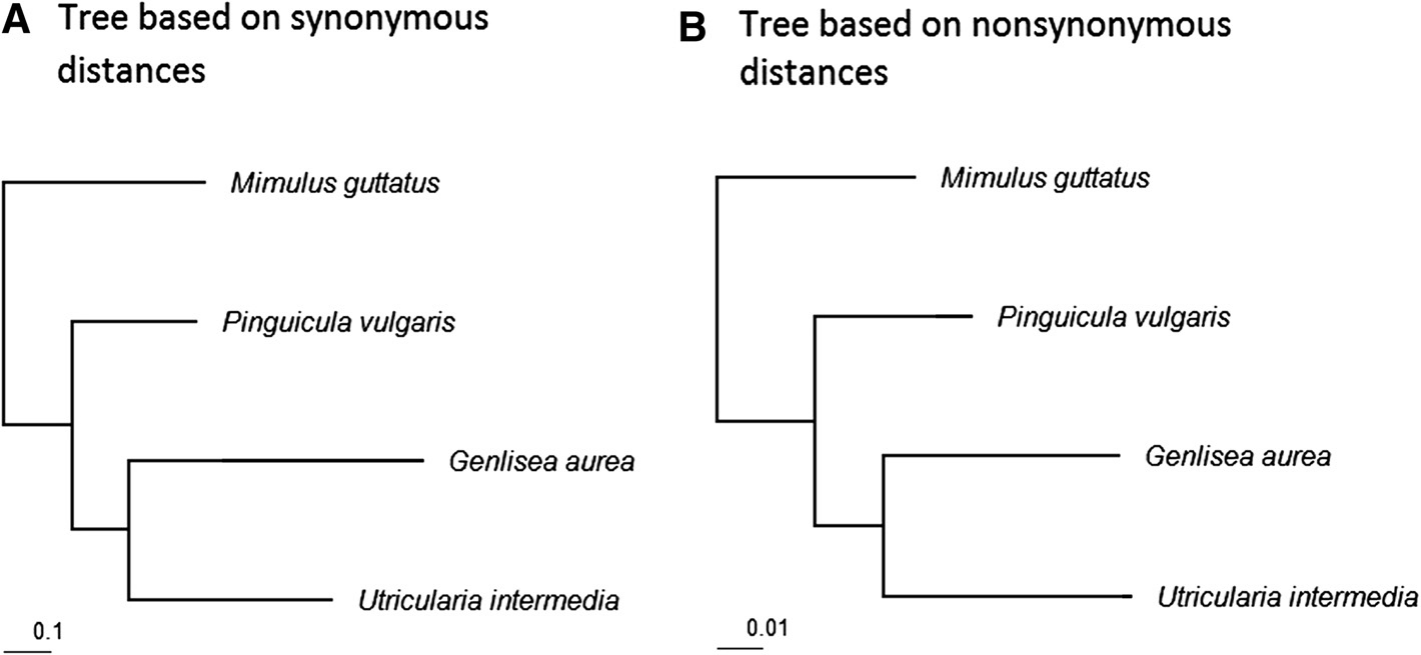
**Phylogenetic trees for *Genlisea aurea *and related species based on synonymous (A) and non-synonymous (B) substitutions.**

According to previous observations, *Genlisea* and *Utricularia* have some of the highest rates of evolution in angiosperms [19,29]. Possible explanations of this fact are relaxed selection in this clade [20] and increased mutation rate due to reactive oxygen species [22]. We calculated evolutionary distances at synonymous and nonsynonmous sites between *G. aurea*, *U. intermedia*, *P. vulgaris*, and *M. guttatus* using PAML v.5.0, with *Solanum lycopersicum* used as an outgroup to root the tree (Figure 5). Distances in the two trees are mostly proportional, and dN/dS ratio for *G. aurea*, *U. intermedia*, *P. vulgaris*, and *M. guttatus* lineages are 0.08, 0.12, 0.13, and 0.10 respectively. Thus, we see no evidence of reduced stringency of selection in the *Genlisea* + *Urticularia* clade.

## Discussion and conclusions

A number of features of the smallest known angiosperm genome of *Genlisea aurea* are worth mentioning. First, the GC-content of *G. aurea* genome is highly variable along its length. This is likely due to non-uniform recombination rate, which can affect GC-content due to weak selection and/or biased gene conversion. Indeed, GC-content is higher in regions with high recombination rates in both metazoa [30-32] and in plants [33,34]. Negative correlation of intron length with GC-content is also considered to be the signature of variable recombination rates [35,36]. The most striking characteristic of *G. aurea* genome is that it contains low number of genes. Although we are unable to report the absolute number of genes because our assembly does not cover the genome completely, results of the search of core eukaryotic genes and of the assembly test demonstrate that we could have missed not more than 10-20 % of genes. This gives an upper estimate of gene number ~ 21 thousand – much lower than is known for any other angiosperm. Sterck et al. [37] hypothesize that ancestral angiosperms could have much less genes than the recent ones, about 12–14 thousands. However, an early-branching and morphologically primitive angiosperm *Amborella trichopoda* has a standard angiosperm gene number, about 27 thousands (amborella.org). In non-flowering plants gene number is also higher: 22–35 thousand [38-40]. Thus we believe that 12–14 thousands is an underestimate. A low gene number in *G. aurea* is even more surprising because this species, as well as all Lentibulariaceae species, is carnivorous. Indeed, one may expect this adaptation to depend on a number of specialized proteins. However, if carnivory results from the modification of existing metabolic pathways, instead of the appearance of the new pathways [41,42], this expectation is wrong and carnivory can evolve without any expansion of the gene repertoire. *G. aurea* genome is one of the first characterized genomes from carnivorous plants (during revision of this manuscript the article reporting the genome of another carnivorous plant, *Utricularia gibba*, was published [43]), and data on more such genomes and their comparative analysis would help to reveal molecular mechanisms of carnivory.

Besides reduction of gene number, we found that both introns and intergenic regions in the *G. aurea* genome are unusually short. In contrast, the per gene number of introns is typical for an angiosperm. Thus, the reduction of genome size in the *G. aurea* lineage was due to both gene loss and non-coding sequences shrinking, but not to intron loss. In all studies performed so far, angiosperm genome reduction not preceded by recent WGD was found to be caused by the loss of non-coding genome segments, including transposable elements [18], and no substantial decrease in gene number has been observed. The exact mechanisms and timing of such decrease are however still unknown – the gene loss or pseudogenization could have occurred in large-genome *Genlisea* ancestor, and small-genome *Genlisea* lineage could have lost pseudogenes and other non-coding genome segments. The study of closest large-genome relatives of small-genome *Genlisea* species is necessary to test this possibility. By now the closest to *G. aurea* species with a known genome is *Mimulus guttatus*. Because the evolutionary distance between these two genomes is substantial (Figure 5), we do not know if gene loss in the *G. aurea* lineage involved pseudogenization followed by slow shrinking of pseudogenes similar to that observed in *Mycobacterium leprae*[44] or occurred through long deletions. Both scenarios can occur only for genes which became functionally redundant. Because the reduction of the genome size of *G. aurea* occurred rapidly, it was likely driven by selection, instead of deletion bias in the mutation process that is thought to be one of the major determinants of genome size [45]. There is an increasing evidence of that genome size is not only due to mutation bias but can also be affected by selection [46,47]. Genome size is correlated with a variety of morphological traits such as seed mass [48], cell size and stomatal density [49]. Correlations between genome size and generation time and mating system are also widely discussed but are less clear. Annuals usually have smaller genomes than perennials (reviewed in [50]) though in these latter there is wider range of genome sizes that overlaps with that of annuals [51,52]. Also, many outcrossing species were reported to have larger genomes than their selfing relatives [53,54] however recent broad-scale comparative analysis suggests that phylogenetic signal could substantially affect this correlation [55]. Vinogradov [56,57] demonstrated, on both plants and animals, that threatened species tend to have larger genomes than their secure relatives (i.e. there is a correlation between the genome size and likelihood of extinction); and vice versa, reduction of genome size correlates with the invasive ability [58]. On the other hand, the reduction of genome size could reduce phenotypic plasticity [59].

There are two mechanisms that are thought to be major driving forces of genome reduction, unequal homologous recombination and illegitimate recombination [60,61]. But irrespectively of the mechanism, sequences under negative selection are unlikely to be lost. Thus, one may expect the genome contraction to proceed primarily through the removal of non-functional regions (sometimes referred to as “junk DNA”). One possible cause of such removal may be an increase of the effective population size, assuming that getting rid of “junk” DNA is advantageous [62]. However in plants the relationship between genome size and effective population size is yet to be clarified [55]. The increased strength of selection favoring reduced genome size is another possibility. Recent study of the genome size change in the genus *Arabidopsis* demonstrates that long (>5 bp) deletions are selectively favored in *A. thaliana*[18]. A similar process could be driving the genome contraction in *G. aurea*. As already mentioned above, short life cycle and self-pollination are important factors in the reduction of genome size. *G. aurea* is a perennial plant [63]; as for the breeding system, though insect visitation of *G. aurea* flowers is documented, there are no direct evidences of cross-pollination. The results of crossing experiments on *Genlisea* species grown in cultivation demonstrated that some members of the genus are facultative autogamous [64]. If *G. aurea* is self-pollinated, in contrast to its large-genome relatives, this transition to selfing could have contributed into the reduction of genome size.

While this paper was in review, a description of the 82 Mb genome of *Utricularia gibba* has been published [43]. There are several common features between this genome and that of *G. aurea*, although they underwent miniaturization independently. In particular, both genomes have a reduced number of protein-coding genes, and the gene densities (28 per 82 in *U. gibba* vs. 21 per 64 in *G. aurea*) are rather similar, suggesting that there may be a minimal complement of non-coding sequences (1,500 nucleotides) per gene in angiosperms.

## Methods

### Origin, cultivation, sequencing and assembly

The plants were cultivated in the private collection of carnivorous plants (A. Seredin, Moscow, Russia). Before DNA extraction, plants were put into distilled water and starved for two days. Total genomic DNA was extracted using modified CTAB-method [65]. To construct the libraries for whole genome sequencing DNA was processed as described in the TruSeq DNA Sample Preparation Guide (Illumina). Two libraries with average length of 413 and 623 bp were selected for sequencing. Libraries were quantified using fluorimetry with Qubit (Invitrogen, USA) and real-time PCR and diluted up to final concentration of 8 pM. Diluted libraries were clustered on two lanes (one library per lane) of a paired-end flowcell using cBot instrument and sequenced using HiSeq2000 sequencer with TruSeq SBS Kit v3-HS (Illumina, USA). Raw reads in fastq format (about 347 millions in total) were imported into CLC Genomic Workbench program; after trimming of adapter-derived and low (Q-score below 30) quality sequences they were assembled using built-in de novo assembly application (k-mer size 64, bubble size 1,000). To minimize the presence of contigs derived from contamination (symbiotic bacteria, prey organisms) we have taken for subsequent analysis only the contigs with average coverage more than 75×. Due to several limitations RNA extraction from *G. aurea* itself was not feasible with the material that we had. Thus to improve the annotation of its genome we sequenced transcriptomes of two related species, *Pinguicula vulgaris* and *Utricularia intermedia*. The samples were taken from Moscow State University botanical garden. Total RNA was extracted from leaves using Qiagen RNEasy Plant Mini kit. About 1 microgram of total RNA was processed using TruSeq RNA Sample Preparation Guide (Illumina). Libraries were sequenced on a single-read flowcell with the read length 100 bp using HiSeq2000 instrument and TruSeq SBS Kit v3-HS (Illumina). Reads were trimmed and assembled using CLC Genomics Workbench 5.0.1 with word size = 36 and bubble size = 2,500.

### Taxonomic filtering for contamination

Contigs with read coverage greater than 75 were compared against the nt database (downloaded on December 29, 2011) using translated-query translated-databased BLAST (tblastx) with e-value cutoff 10^-6^ and default options otherwise. Contigs were selected as passing the taxonomy filter if their top TBLASTX hit (according to e-value) belonged to *Magnoliophyta*, or they had no TBLASTX hits with this cutoff.

### Nuclear genome annotation

Assembled contigs were subject to repeat identification using program RepeatMasker (v. open-3.3.0, [66]) using Embryophyta repeats (6.0% of genome were masked) and de novo repeat search tool RepeatScout [67] with default parameters except “--thresh = 10” for “filter-stage-2” step (additional 7.2% of genome were masked giving 5,722,364 bp out of 43,366,824 bp in total). After repeat masking, contigs were subject to independent gene prediction with four different approaches. First, we used GENEWISE [68] to predict genes in loci which are similar to *Mimulus guttatus*, *Arabidopsis thaliana*, and *Solanum lycopersicum* genes and separately to all Uniprot proteins. Similarity was detected with BLASTX of *Genlisea aurea* contigs against predicted proteomes of these organisms/Uniprot proteomes. In the cases of overlapping predictions the longest gene was taken. Second, we used transcribed sequences of closely related species *Utricularia intermedia* and *Pinguicula vulgaris* to predict genes in *Genlisea aurea* with GENESEQER [69]. Third, we performed ab initio similarity based gene prediction with AUGUSTUS [70] v.2.5.5. with gene model trained on 94 genes selected from genes predicted by previous two methods. Each selected gene should have similar protein in Uniprot with 95% coverage of amino acid sequences of both proteins by hit region. “--UTR = off” parameter was used for training procedure. As an input data for prediction by Augustus the hint-files were made using BLAT for alignment on the genome contigs the data from 454 transcriptome sequencing of *Utricularia gibba*, the species from sister genus *Utricularia* ([21], SRA accession number SRR094438) and proteins from 20 plant genomes available in PlantGDB [10] on March 2012. These are *Arabidopsis thaliana* (annotation version TAIR10), *Brachypodium distachyon* (192), *Brassica rapa* (197), *Carica papaya* (113), *Chlamydomonas reinhardtii* (169), *Cucumis sativus* (JGI1.0), *Glycine max* (109), *Lotus japonicus* (Kazusa1.0), *Manihot esculenta* (147), *Mimulus guttatus* (140), *Oryza sativa* (MSU7.0), *Physcomitrella patens* (152), *Prunus persica* (139), *Populus trichocarpa* (156), *Sorghum bicolor* (79), *Setaria italica* (164), *Solanum lycopersicum* (ITAG2), *Selaginella moellendorffii* (91), *Volvox carteri* (199), *Zea mays* (5b.60). Fourth, ab initio self-training gene prediction method GeneMark-ES [71] was used with parameter “--min_contig 10000”.

Finally, we took the union of these seven annotations (1 – AUGUSTUS, 4 – GENEWISE, 2 – GENESEQER, 1 - GeneMark-ES). In the case of overlapping genes we took one with the best hit in Uniprot or in *M. guttatus*, *A. thaliana*, *S. lycopersicum*, or *O. sativa*. If there was no significant hit for any of overlapping predictions, we took the longest one. Number of genes predicted with each program and overall number of genes in the final set are listed in Table 1. To search Pfam-domains all predicted genes were scanned with PfamScan on database Pfam-A v.26 [72]. To identify the clusters of orthologous genes, OrthoMCL [73] was run on five plant genomes: *G. aurea, M. guttatus, A. thaliana, S. lycopersicum*, *O. sativa*.

To perform Gene Ontology annotation we used BLAST2GO [9]. Using predicted transcript set as a query BLAST was run with the e-value cut-off 10^-3^ and the annotation with e-value cut-off 10^-5^. To provide a summary of the results of GO annotation of *Genlisea* genome plant GO-slim categories developed by TAIR were used. GOstat [74] was used to find statistically overrepresented and underrepresented gene ontologies.

### Test of the assembly and gene number estimates

The data on *Arabidopsis* genome were generated using the same experimental protocols as those of *Genlisea*. 50 millions of paired 100 bp reads were used for assembly. Two datasets were used: “clean” – containing only *Arabidopsis* reads and “contaminated”. To generate the latter, we performed a back-mapping of all reads used for assembly of *Genlisea* genome on filtered contigs (mapping parameters: aligned length 0.8, similarity 0.95) and collected the reads that did not map. These reads were added to *Arabidopsis* sequence data and assembled. Assembly was performed using CLC Genomics Workbench 6.0 with the same parameters as for assembly of *Genlisea* genome; the same was done with clean Arabidopsis reads. After assembly we analyzed coverage distribution for the assemblies of clean and contaminated datasets and removed from the contaminated assembly contigs with low coverage (less than 40×). Then contigs of both datasets were aligned on reference chromosomes of TAIR10 assembly by BLAT. All hits shorter than 1,000 bp (either in contigs or in chromosomes) and with identity below 90% were removed. All matched regions in reference chromosomes were joined to avoid hit overlapping. After that genes of reference annotation were tested for coverage by contigs of both datasets. Gene was classified as covered in case at least 50% of it was covered.

### Calculating phylogenetic distances

For each *G. aurea* gene we searched with BLAST for the best homologous gene in *M. guttatus* and *S. lycopersicum* genomes and *U. intermedia* and *P. vulgaris* transcriptomes. We obtained 8,677 groups of homologous genes, which are present in each of these 5 organisms. Then, each group of homologous genes was aligned with MACSE [75]. Finally, the concatenate of alignments was used to calculate synonymous and nonsynonymous distances with codeml program from PAML package [76]. Only codon columns present in each 5 species were used in the analysis, *S. lycopersicum* sequence was used to root the tree.

### Data access

Annotated genome of *G. aurea* is available in the Genbank under BioProject accession number PRJNA208769. Data from other species generated in this study are available under BioProject accession number PRJNA211836.

## Competing interests

The authors declare they have no competing interests.

## Authors’ contributions

EVL carried out computational analysis and participated in writing, RAS annotated the assembly and carried out OrthoMCL analysis, ERN carried out filtering of contigs, AAP constructed transcriptome libraries, participated in sequencing and assembled contigs, ASK participated in the design and coordination of the study and contributed to manuscript preparation, MDL constructed and sequenced DNA libraries, conceived and coordinated the study and drafted the manuscript. All authors read and approved the final manuscript.

## Supplementary Material

Additional file 1 Distribution of contigs by their coverage. Click here for file

Additional file 2 **Ten top blast hits for predicted transcripts of *Genlisea aurea *and transcriptome assembly of its relatives *Pinguicula vulgaris *and *Utricularia intermedia***. Click here for file

Additional file 3 Frequency of contigs with a given GC-content. Frequencies predicted under the assumption of uniform distribution of GC-nucleotides are shown in orange, those that are observed in the assembly are shown in grey. Click here for file

Additional file 4 **Distribution of intron lengths in *Genlisea aurea *genome.** Click here for file

Additional file 5 **Numbers (A) and fractions (B) of genes in the aligned blocks for each gene family size. **On each panel, the full length of the bar indicates the number of genes in *M. guttatus* genome and the filled part of the bar indicates the number of genes in *Genlisea aurea* genome. The empty part of the bar corresponds to genes which are presumably lost in in *Genlisea aurea*. Click here for file

Additional file 6 **Analysis of GO-enrichment of *Mimulus *genes that have ortholog in *Genlisea *genome. **Underrepresented GO categories are shown in red and overrepresented in green. Click here for file
