# Supplementary figures and images for: The miniature genome of a carnivorous plant Genlisea aurea contains a low number of genes and short non-coding sequences
Source: BMC Genomics. 2013 Jul 15;14:476. doi: 10.1186/1471-2164-14-476 (PMC3728226; doi:10.1186/1471-2164-14-476)

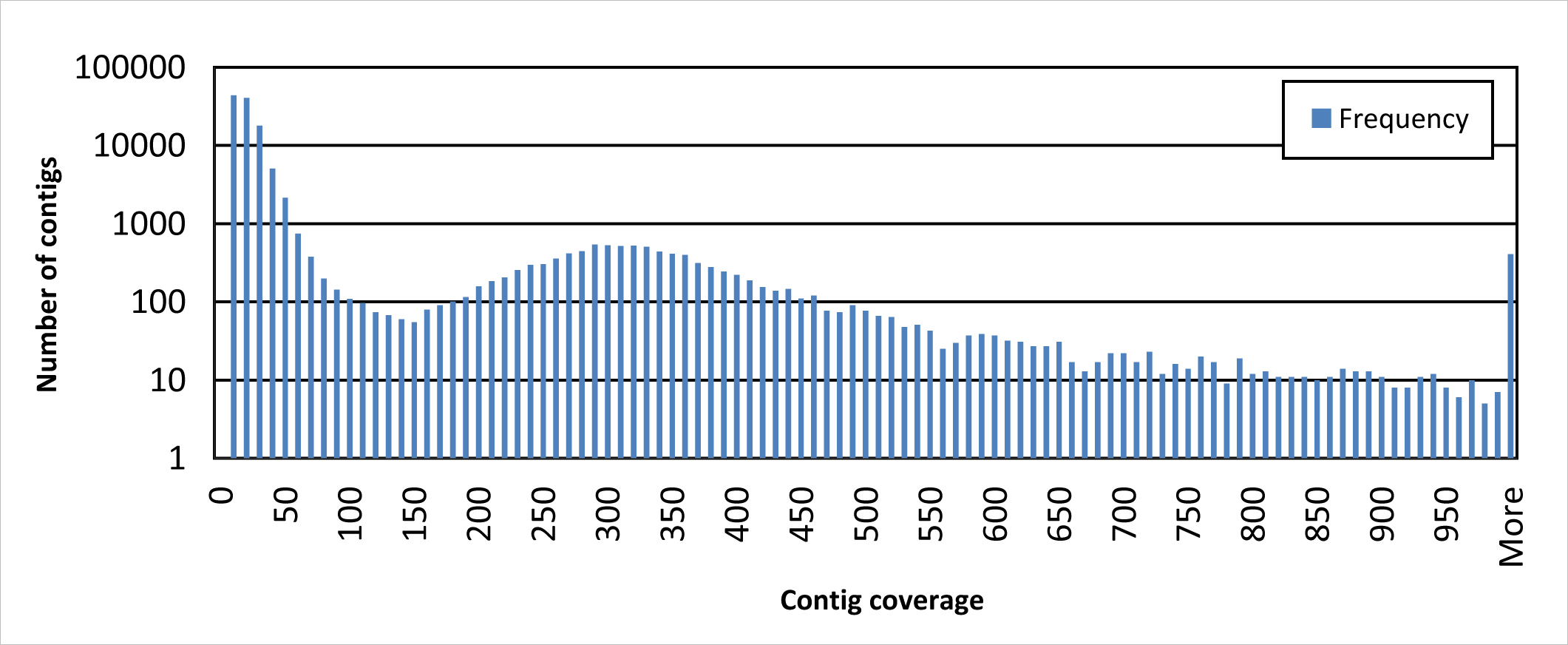

Supplement: Additional file 1 — Distribution of contigs by their coverage. [file 1471-2164-14-476-S1.jpeg]

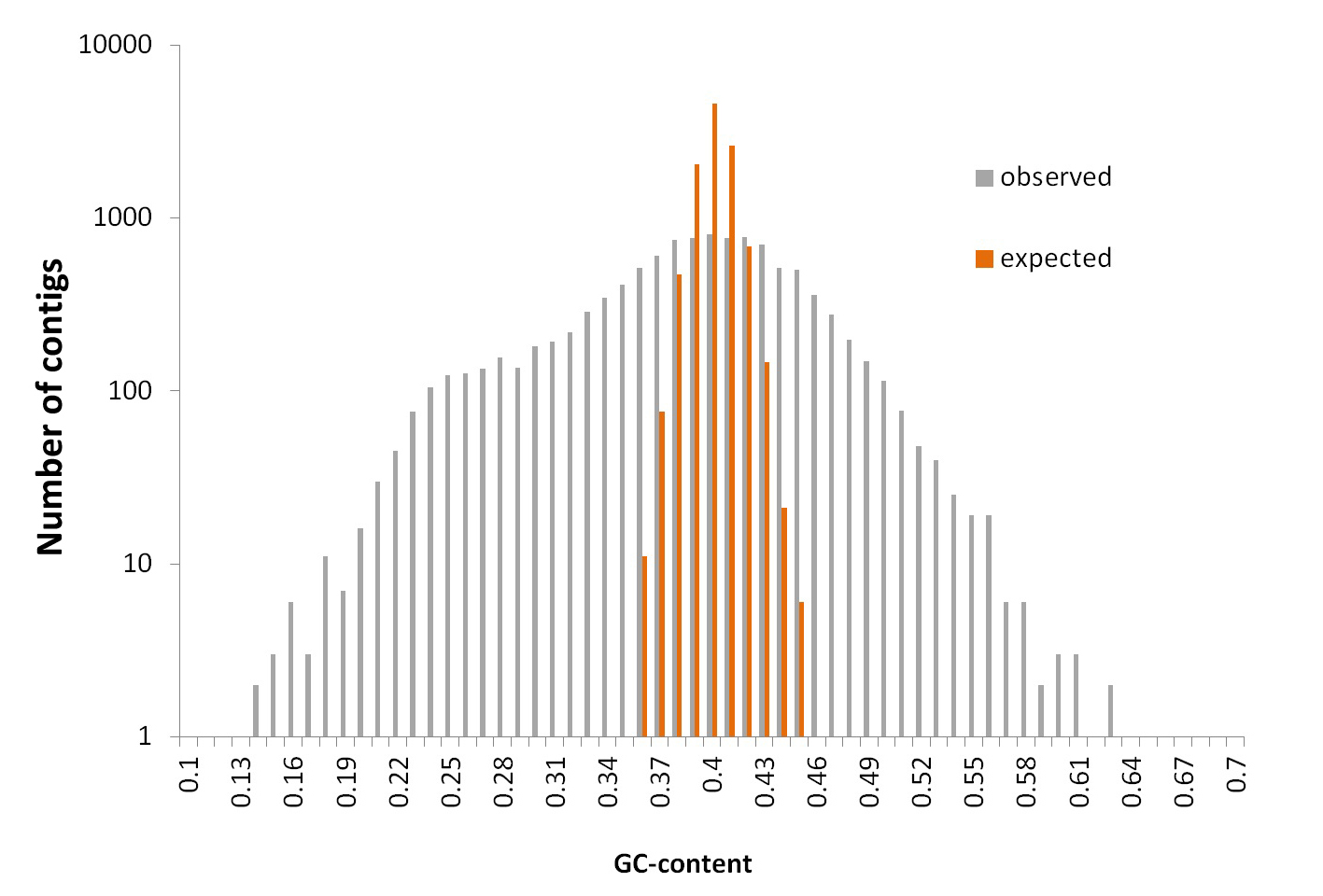

Supplement: Additional file 3 — Frequency of contigs with a given GC-content. Frequencies predicted under the assumption of uniform distribution of GC-nucleotides are shown in orange, those that are observed in the assembly are shown in grey. [file 1471-2164-14-476-S3.jpeg]

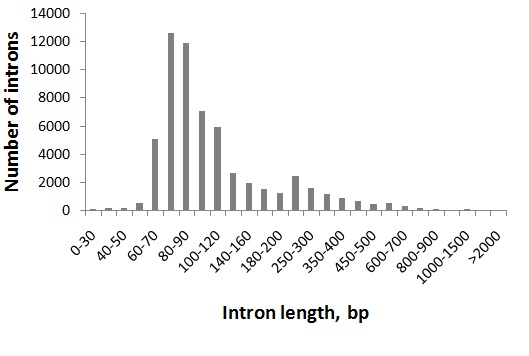

Supplement: Additional file 4 — Distribution of intron lengths in Genlisea aurea genome. [file 1471-2164-14-476-S4.jpeg]

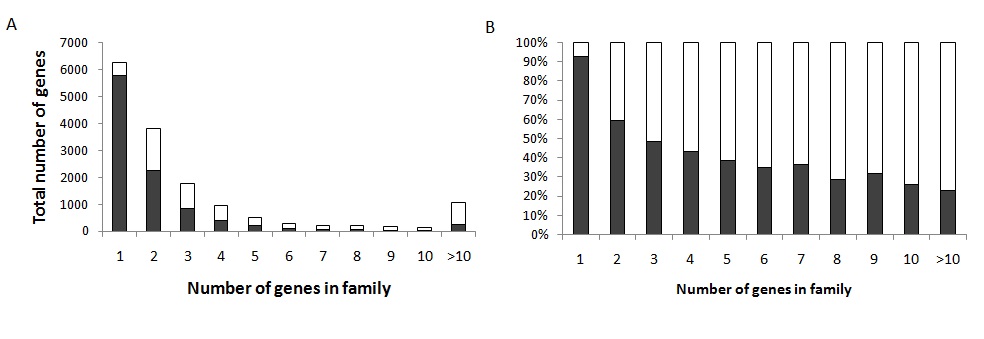

Supplement: Additional file 5 — Numbers (A) and fractions (B) of genes in the aligned blocks for each gene family size. On each panel, the full length of the bar indicates the number of genes in M. guttatus genome and the filled part of the bar indicates the number of genes in Genlisea aurea genome. The empty part of the bar corresponds to genes which are presumably lost in in Genlisea aurea. [file 1471-2164-14-476-S5.jpeg]
