# Supplementary material for: The miniature genome of a carnivorous plant Genlisea aurea contains a low number of genes and short non-coding sequences
Source: BMC Genomics. 2013 Jul 15;14:476. doi: 10.1186/1471-2164-14-476 (PMC3728226; doi:10.1186/1471-2164-14-476)
Supplement: Additional file 2 — Ten top blast hits for predicted transcripts of Genlisea aurea and transcriptome assembly of its relatives Pinguicula vulgaris and Utricularia intermedia. [file 1471-2164-14-476-S2.doc]

| Pinguicula | | Uticularia | | Genlisea | |
| --- | --- | --- | --- | --- | --- |
| Species | BLAST Top-Hits | Species | BLAST Top-Hits | Species | BLAST Top-Hits |
| Vitis vinifera | 11731 | Vitis vinifera | 12395 | Vitis vinifera | 4515 |
| Populus trichocarpa | 4313 | Populus trichocarpa | 4879 | Ricinus communis | 1527 |
| Ricinus communis | 3983 | Ricinus communis | 4576 | Populus trichocarpa | 1504 |
| Glycine max | 2641 | Glycine max | 3478 | Glycine max | 1057 |
| Medicago truncatula | 968 | Medicago truncatula | 1285 | Medicago truncatula | 352 |
| Nicotiana tabacum | 701 | Arabidopsis thaliana | 1055 | Arabidopsis thaliana | 288 |
| Arabidopsis thaliana | 677 | Nicotiana tabacum | 743 | Solanum lycopersicum | 255 |
| Solanum lycopersicum | 547 | Arabidopsis lyrata | 650 | Nicotiana tabacum | 241 |
| Arabidopsis lyrata | 430 | Solanum lycopersicum | 634 | Arabidopsis lyrata | 214 |
| Lotus japonicus | 359 | Oryza sativa | 462 | Oryza sativa | 146 |
